# A Case of Obturator Hernia Involving the Urinary Bladder Discovered Following a Femoral Incarcerated Hernia

**DOI:** 10.70352/scrj.cr.25-0097

**Published:** 2025-04-21

**Authors:** Nobuhiro Naito, Toshiki Hirakawa, Mikio Nambara, Naoki Kametani, Akiko Tachimori, Nobuya Yamada, Shigehiko Nishimura, Naoyuki Taenaka

**Affiliations:** Digestive Surgery Department, Sumitomo Hospital, Osaka, Osaka, Japan

**Keywords:** strangulated femoral hernia, obturator hernia, bladder hernia, TAPP

## Abstract

**INTRODUCTION:**

Obturator hernias are rare, accounting for 0.05%–1.4% of all hernias, and typically affect elderly, thin women. Bladder hernias, though uncommon, comprise 1%–4% of groin hernias, with bladder herniation through the obturator foramen being extremely rare. We report a case of an obturator hernia involving the urinary bladder, which was incidentally discovered during femoral hernia repair.

**CASE PRESENTATION:**

A 70-year-old woman presented with a 2-day history of abdominal pain and vomiting. She had no urinary symptoms. Abdominal computed tomography (CT) revealed a right femoral hernia and an unexpected bladder herniation through the obturator foramen. Laparoscopic transabdominal preperitoneal (TAPP) repair was performed using 3 ports. The incarcerated bowel was reduced after incising the lacunar ligament. The prolapsed bladder was carefully dissected to prevent injury, and a dual-layered Bard mesh (Medicon, Franklin Lakes, NJ, USA) was placed to reinforce the defect. The patient recovered uneventfully and was discharged on the 7th postoperative day. No recurrence or urinary symptoms were observed several months postoperatively.

**CONCLUSIONS:**

Bladder herniation through the obturator foramen is extremely rare and often asymptomatic, making preoperative diagnosis challenging. Surgeons should consider this condition during hernia repair to prevent intraoperative bladder injury. Preoperative imaging is crucial for safe and complete surgical management.

## INTRODUCTION

Obturator hernias account for only 0.05%–1.4% of all hernias and most commonly affect thin, elderly women. These hernias are often diagnosed following small bowel obstruction. Bladder hernias, though rare, account for approximately 1%–4% of groin hernias in adults. However, few cases of bladder hernias through the obturator foramen have been reported. In this report, we describe a case of an obturator hernia involving the urinary bladder, discovered incidentally during a femoral hernia repair, along with a discussion of the relevant literature.

## CASE PRESENTATION

### Patient

A 70-year-old female.

### Chief complaints

Abdominal pain and vomiting.

### History of present illness

The patient was admitted to our hospital with complaints of abdominal pain and vomiting for the past 2 days. No urinary symptoms, such as dysuria or urinary frequency, were noted.

### Past medical history

Unremarkable.

### Medication history

None of note.

### Vital signs on admission

Height: 165.4 cm; weight: 68.9 kg; BMI: 25.3 kg/m^2^; Body temperature: 36.2°C; blood pressure: 112/77 mmHg; pulse: 72 bpm; respiratory rate: 16 breaths/min.

### Abdominal examination

Mild abdominal distention, soft on palpation. There was no peritoneal irritation, although spontaneous pain was noted throughout the abdomen.

### Laboratory findings

Mild elevation of inflammatory markers, with no other notable findings.

### Abdominal computed tomography (CT) findings (Fig. 1)

A right femoral hernia was identified. No areas of poor contrast were observed in the bowel. The bladder was noted to herniate into the right obturator foramen. Based on these findings, the patient was diagnosed with a right femoral hernia and a right obturator bladder hernia. Surgical repair was planned.[Fig F1]

**Fig. 1 F1:**
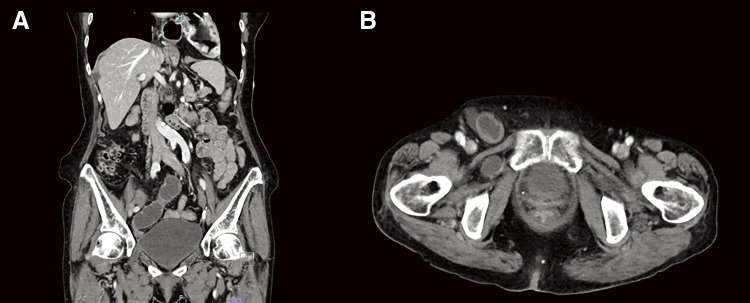
Contrast-enhanced abdominal CT scan (**A**: coronal section; **B**: axial section). A right femoral hernia was found. There was no area of poor contrast in the mated intestine. The bladder was located in the right obturator foramen. CT, computed tomography

### Surgical procedure (Fig. 2)

Laparoscopic surgery was performed using 3 ports: a 12 mm camera port at the umbilicus and two 5 mm ports in the bilateral flanks. No hernia was noted on the contralateral side. Despite attempts to reduce the incarcerated small bowel with traction and hydrostatic pressure, the hernia could not be reduced. However, once the lacunar ligament was incised, the bowel was successfully released.[Fig F2]

**Fig. 2 F2:**
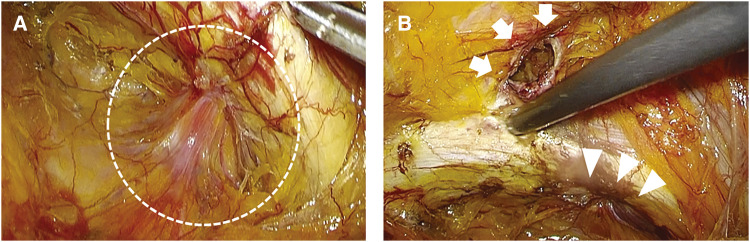
(**A** and **B**) Surgical findings. (**A**) The bladder was incarcerated in the obturator foramen (circled). (**B**) After exposing the MPO, the femoral ring (arrow) and obturator foramen (arrowhead) were identified. MPO, myopectineal orifice

The hernia defect was repaired using the transabdominal preperitoneal (TAPP) approach. Prolapsed bladder was observed in the obturator foramen, and careful dissection was performed to avoid bladder injury. The myopectineal orifice (MPO) was exposed, revealing a 2 × 2 cm defect on the dorsal side of the iliopubic tract. The patient was diagnosed with a right femoral hernia (F2 type according to the 2021 New JHS classification) and a right obturator bladder hernia.

A Bard mesh (Medicon, Franklin Lakes, NJ, USA) was trimmed to 2 pieces (13 × 10 cm and 13 × 7 cm) and layered to cover the defect. The first mesh was place to cover the closure hole and the femoral ring. Since the posterior wall of the inguinal canal was then not covered with mesh, another piece of mesh was placed on top and bottom to cover the posterior wall of the inguinal canal. The mesh was tacked in place using OptiFix (Medicon) at 3 locations: medial and lateral to the inferior abdominal wall arteriovenous vein and at Cooper’s ligament. The peritoneum was closed with continuous sutures using 3-0 V-Loc (Medtronic, Minneapolis, MN, USA). No ischemic changes were noted in the incarcerated bowel, so no bowel resection was performed. The total operative time was 1 hour and 37 minutes with minimal blood loss.

### Postoperative course

The patient started drinking fluids on the 1st postoperative day and eating on the 3rd day. The recovery was uncomplicated, and the patient was discharged on the 7th postoperative day. Several months after surgery, there has been no recurrence of the hernia, and the patient has not reported any urinary symptoms.

## DISCUSSION

Obturator hernias are a rare condition, comprising only 0.05%–1.4% of all hernias.^1)^ Bladder hernias, in which part or all of the bladder protrudes through an abdominal or pelvic opening, are also uncommon, accounting for 1%–4% of inguinal hernias.^2)^ Most bladder hernias occur as indirect inguinal hernias, while those occurring via the obturator foramen are exceedingly rare.^3)^

A search of the Japan Medical Abstracts Society (Ichushi) database using the keywords “bladder hernia” and “obturator hernia” for publications from 1983 to December 2024, excluding conference abstracts, identified only 8 cases of bladder hernia with the obturator foramen as the hernial orifice in Japan, including our own case^4–10)^ (**Table 1**). Among these cases, 4 were right-sided, 3 left-sided, and 1 was bilateral. The mean patient age was 78 years (range: 66–96 years), and the male-to-female ratio was 1:7, indicating a higher prevalence in women.

**Table 1:** Reported cases of bladder hernia with the obturator foramen as the hernial orifice in Japan

	Author	Year of report	Age	Sex	Affected side	Urological symptoms	Classification	Surgical procedure
1	Kikkawa et al.^17)^	2009	96	Female	Right	—	—	—
2	Ogata et al.	2012	80	Female	Both	—	—	—
3	Watanabe et al.	2016	77	Female	Left	—	Paraperitoneal type	Open
4	Kuge et al.	2017	74	Male	Right	—	Paraperitoneal type	TAPP
5	Inoue et al.	2018	78	Female	Right	—	Paraperitoneal type	Open
6	Yuba et al.	2019	79	Female	Left	—	Paraperitoneal type	TEP
7	Kuhara et al.	2023	66	Female	Left	+	Extraperitoneal type	TAPP
8	Present case	2025	70	Female	Right	—	Extraperitoneal type	TAPP

TAPP, transabdominal preperitonea; TEP, totally extraperitoneal.

Funyu et al. proposed the following factors contributing to the development of bladder hernia: (1) congenital or acquired weakening of the abdominal wall, (2) congenital abnormalities of the bladder wall or bladder muscle, (3) bladder distension and relaxation due to lower urinary tract obstruction (e.g., prostatic hypertrophy and bladder neck sclerosis), (4) accumulation of adipose tissue in the prevesical space, (5) increased bladder pressure, and (6) obesity.^11)^

**Fig. 3:**
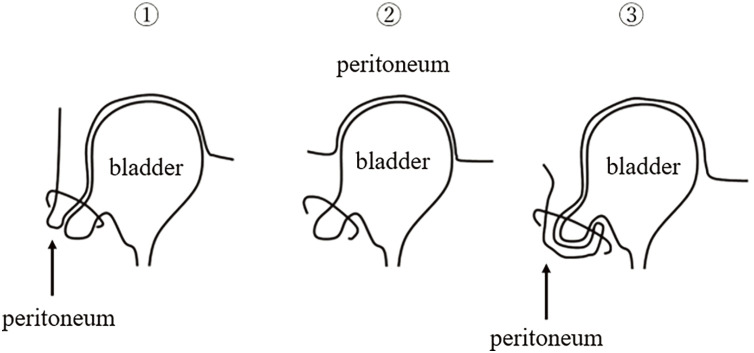
Classification. ① paraperitoneal type (both bladder and peritoneum herniate), ② extraperitoneal type (only bladder herniates), and ③ intraperitoneal type (bladder herniates with peritoneal covering).

Bladder hernias are classified into 3 types based on the anatomical relationship between the herniated bladder and the peritoneum (**Fig. 3**): (1) paraperitoneal type (both bladder and peritoneum herniate), (2) extraperitoneal type (only bladder herniates), and (3) intraperitoneal type (bladder herniates with peritoneal covering).^12)^ In Japan, the paraperitoneal type is most common (62.2%), followed by the extraperitoneal type (35.1%) and the intraperitoneal type (2.7%).^13)^ Among reported cases of bladder hernia via the obturator foramen, 66.7% were paraperitoneal and 33.3% were extraperitoneal.

In our case, the peritoneum was not involved, and only the bladder herniated into the femoral ring, indicating an extraperitoneal bladder hernia. As extraperitoneal bladder hernias may not present as visible depressions on the peritoneal side, they can be overlooked, highlighting the importance of preoperative imaging for accurate diagnosis.

Symptoms of bladder hernias often include urinary disturbances such as dysuria, 2-stage urination, and frequent urination.^13)^ Diagnostic methods include ultrasound, CT, and cystography. However, smaller hernias may not cause noticeable urinary symptoms, and only around 23% of patients experience 2-stage urination, making preoperative diagnosis challenging.^14)^ Among the 8 reported cases of bladder hernia via the obturator foramen in Japan, only 1 exhibited urinary symptoms, further suggesting the difficulty of preoperative diagnosis.

Kraft et al. reported that fewer than 7% of bladder hernias are diagnosed preoperatively, while 16% are diagnosed postoperatively due to complications like bladder injury, and the rest are diagnosed intraoperatively.^15)^ While preoperative diagnosis is essential to avoid bladder injury, bladder hernias can coexist with inguinal hernias without causing urinary symptoms.^16)^ Surgeons should consider the possibility of a bladder hernia during inguinal hernia repair, particularly in patients with urinary symptoms. Preoperative imaging, including ultrasound and CT, is recommended to ensure a safe surgery.

## CONCLUSION

Preoperative identification of hernial contents can help avoid organ injury during surgery and contribute to safer and more complete surgical procedures.

## DECLARATIONS

### Funding

The authors have no funding to report.

### Authors’ contributions

All authors contributed to the conception, design, and execution of the research, as well as the analysis and interpretation of data.

They were also involved in drafting and revising the manuscript.

All authors have read and approved the manuscript.

### Availability of data and materials

The data and materials used in this case report are not publicly available due to patient privacy concerns.

### Ethics approval and consent to participate

This work does not require ethical considerations or approval. Informed consent to participate in this study was obtained from the patient.

### Consent for publication

The patient has provided consent for this case report to be published.

### Competing interests

The authors declare that they have no competing interests.
